# Subnational health management and the advancement of health equity: a case study of Ethiopia

**DOI:** 10.1186/s41256-019-0105-3

**Published:** 2019-05-17

**Authors:** Nicole Bergen, Arne Ruckert, Manisha A. Kulkarni, Lakew Abebe, Sudhakar Morankar, Ronald Labonté

**Affiliations:** 1 University of Ottawa, 600 Peter Morand Crescent, Ottawa, ON K1G 5Z3 Canada; 2 Jimma University, PO Box 378, Jimma, Ethiopia

**Keywords:** Case study, Ethiopia, Health equity, Health governance, Health systems, Subnational health managers

## Abstract

**Background:**

Health equity is a cross-cutting theme in the United Nations 2030 Agenda for Sustainable Development, and a priority in health sector planning in countries including Ethiopia. Subnational health managers in Ethiopia are uniquely positioned to advance health equity, given the coordination, planning, budgetary, and administration tasks that they are assigned. Yet, the nature of efforts to advance health equity by subnational levels of the health sector is poorly understood and rarely researched. This study assesses how subnational health managers in Ethiopia understand health equity issues and their role in promoting health equity and offers insight into how these roles can be harnessed to advance health equity.

**Methods:**

A descriptive case study assessed perspectives and experiences of health equity among subnational health managers at regional, zonal, district and Primary Health Care Unit administrative levels. Twelve in-depth interviews were conducted with directors, vice-directors, coordinators and technical experts. Data were analyzed using thematic analysis.

**Results:**

Subnational managers perceived geographical factors as a predominant concern in health service delivery inequities, especially when they intersected with poor infrastructure, patriarchal gender norms, unequal support from non-governmental organizations or challenging topography. Participants used ad hoc, context-specific strategies (such as resource-pooling with other sectors or groups and shaming-as-motivation) to improve health service delivery to remote populations and strengthen health system operations. Collaboration with other groups facilitated cost sharing and access to resources; however, the opportunities afforded by these collaborations, were not realized equally in all areas. Subnational health managers’ efforts in promoting health equity are affected by inadequate resource availability, which restricts their ability to enact long-term and sustainable solutions.

**Conclusions:**

Advancing health equity in Ethiopia requires: extra support to communities in hard-to-reach areas; addressing patriarchal norms; and strategic aligning of the subnational health system with non-health government sectors, community groups, and non-governmental organizations. The findings call attention to the unrealized potential of effectively coordinating governance actors and processes to better align national priorities and resources with subnational governance actions to achieve health equity, and offer potentially useful knowledge for subnational health system administrators working in conditions similar to those in our Ethiopian case study.

## Introduction

Health equity, defined as the absence of avoidable, unfair, or remediable differences in health among subgroups of a population [1, 2], has been widely adopted as a priority for national health sector planning, aligning with commitments such as the United Nations 2030 Agenda for Sustainable Development. The development and implementation of plans to advance health equity, however, have proven to be a complex and difficult undertaking [3, 4]. Meaningfully promoting health equity and addressing the root causes of inequity requires the involvement and coordination of stakeholders across various sectors and levels of governance, each with differing roles and interests [5]. Consequently, the promotion of health equity is beholden to context-specific considerations at national and subnational levels [6].

Ethiopia, a low-income country in east Africa, has made strong national commitments to advance health equity. Equity is a strategic objective in the national Health Sector Transformation Plan (HSTP), which aims to promote “equal access to essential health services, equal utilization [by] equal need, and equal quality of care for all” (p.14), while calling attention to issues of fairness and human rights (noting Ethiopia’s constitutional right to health) [7]. Having a record of substantial – though not necessarily equitable – gains in maternal, newborn and child health (MNCH) during the Millennium Development Goal period (1990–2015) [8], Ethiopia emphasizes equity as a key focus for MNCH and primary health care in particular [9, 10]. For example, the expansion of basic health services into rural areas through the Health Extension Program, which stipulates the provision of publicly-funded MNCH services [11, 12], demonstrates a commitment to improve MNCH outcomes among the rural and economically poor.

Bridging the gap between national-level bureaucrats and the health workforce, subnational health managers are a vital component of health system functioning [13, 14]. Management of health systems – defined as the process of achieving specified objectives through human, financial, and technical resources [15] – is particularly important in low-resource settings, where such resources are in limited supply when juxtaposed against ambitious objectives. For this reason, subnational managers in the health system in Ethiopia (including facility-based managers, as well as those at district (woreda), zonal and regional levels) (Table 1), are uniquely positioned to further national commitments to improve health equity.

**Table 1:** Subnational administrative bodies within the Ethiopia system, regional to local, in the Ethiopian health system

Administrative Level	Name of health administrative unit	Key characteristics and administrative responsibilities
Region	Regional Health Bureau	• Ethiopia has 11 Regional Health Bureaus. • Regional Health Bureaus coordinate and execute all activities in the health sector within the respective region. • Staff at Regional Health Bureaus translate and adapt national guidelines for regional contexts. • Funding is received from the National Ministry of Health (allocated based on population size of the region) and mostly earmarked for specific programs.
Zone	Zonal Health Department	• Ethiopia has approximately 86 zones. • Zonal Health Departments convey guidelines and directives from the region to the woredas. • They provide technical and administrative support to woredas, and monitor their financial performance.
District (Woreda)	Woreda Health Office	• The total number of urban and rural woredas in Ethiopia is more than 800, each with a catchment population of around 100,000. • Woreda-based health sector planning was introduced in HSTP-III (2005/06–2009/10). • Woreda Health Offices (together with PHCU staff) manage health care delivery issues, and perform monitoring and evaluation of health activities in the woreda.
Local: Primary Health Care Unit (PHCU)	PHCU	• A PHCU typically comprises 5 health posts and a health centre. Each PHCU serves a catchment population of about 25,000. • PHCUs employ directors and coordinators who function in management capacities. Management staff at the PHCU have a direct role in overseeing the delivery of care.

Since the early 1990s, the health sector in Ethiopia has been characterized by the process of decentralization and the transfer of decision-making power from national to lower levels of administration [16]. This is evidenced by the HSTP agenda for “woreda transformation”, whereby woreda health offices are largely responsible for the actualization of national governmental priorities such as the delivery of equitable and quality health care [7]. The HSTP additionally recommends that each administrative level develop strategic annual plans that contextualize local priorities and harmonize action under the overarching HSTP. Although decentralization of the health sector is intended to promote equity through improved responsiveness to local needs, some have suggested that, without adequate financial and human resources, clear guidelines, and continuous monitoring, it may exacerbate inequities [17, 18].

Understanding and supporting the advancement of health equity in Ethiopia requires attention to subnational health managers and the variety of coordination, planning, budgetary, and administration tasks that they perform. Indeed, the role of subnational actors in furthering global health initiatives is a growing area of interest in health governance scholarship [13, 19]. Assessments of the characteristics of well-functioning health systems point to several features that resonate with the roles of subnational actors, including: autonomy and flexibility in managing the health system; responsiveness to diverse population needs; strong district health systems that can reach marginalized populations; and engagement with non-state actors and communities [20, 21].

Through a case study in Ethiopia, this article explores the perspectives and experiences of subnational actors as they relate to the advancement of the nationally established priority to improve health equity. The study aims to ascertain how subnational health managers in Ethiopia identify and understand health equity issues and their role in promoting health equity. This empirical exploration is particularly pertinent within the context of Ethiopia, where the roles and responsibilities across different levels of the health system are not always fully understood by those working within the system [22]. Based on our findings, we suggest opportunities to address health equity bottlenecks at subnational levels of the health system, and discuss how efforts, including future research, can be oriented to better understand and advance equity in health. We anticipate that the lessons from this study may have broad applicability in other low- and middle-income countries, where trends of decentralization of the health sector may too create uncertainty surrounding the role of subnational health managers.

## Theorized constructs of health equity

**Fig. 1:**
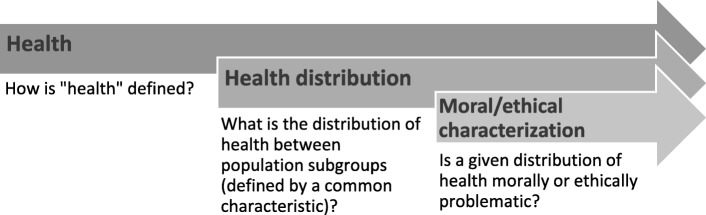
Constructs of health equity

As illustrated in Fig. 1, health equity can be broken down into three constructs: health; distribution of health; and moral or ethical characterization of health distribution. The conceptualization of health may focus on assessments of health status, well-being or functioning [23, 24]; it may also capture any other aspect of the health system (including health governance, health financing, health services access and readiness, and health service coverage) [2, 25], determinants of health, or health-related norms, values, behaviours and attitudes [26]. The distribution of health construct addresses the comparison of health across subgroups, including the question of how subgroups of individuals are defined [27, 28]. The third construct addresses whether a specified aspect of health and its distribution is problematic from a moral/ethical perspective. That is: is the distribution of health fair, i.e. are the health differences between individuals or groups unavoidable? [26]

## Methods

We drew from case study methodology to examine the perspectives and experiences of subnational health managers within one zone of Ethiopia, located in the southwest of the country, and corresponding higher levels of the health system. A descriptive case study design was selected to allow for the holistic exploration of a complex social phenomenon (the advancement of health equity) where the context and phenomenon are not clearly distinct [29]. The findings reported here are part of a larger randomized implementation study across several districts within Ethiopia. Ethics approval for this research was obtained in 2017 (in advance of commencement of data collection) from the University of Ottawa Health Sciences and Science Research Ethics Board and from an Ethiopian University Institutional Review Board. The study was undertaken in compliance with the protocols stated in the ethics approval.

**Fig. 2:**
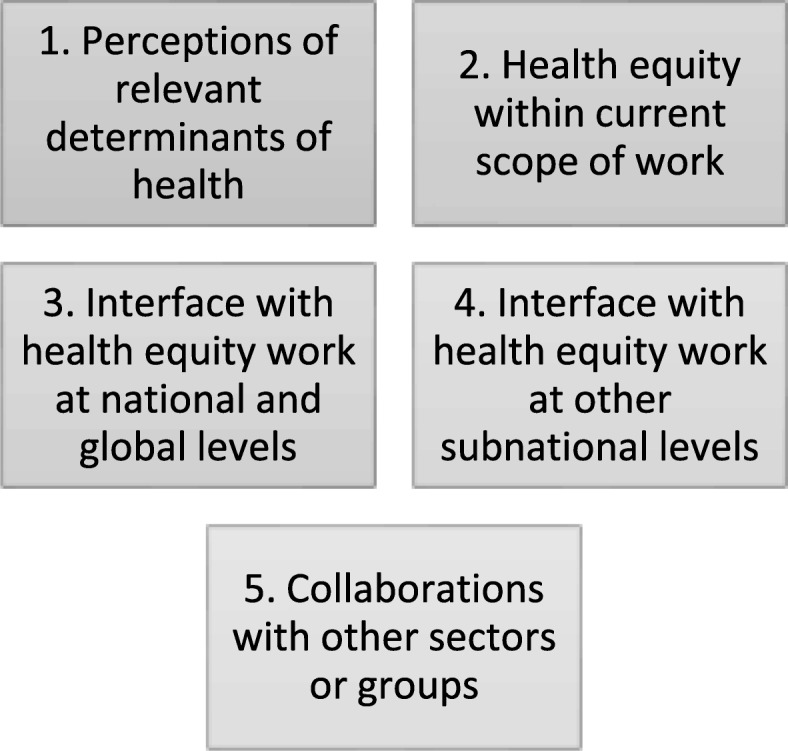
Five domains of investigation in semi-structured key informant interviews with subnational health managers in Ethiopia

Participants were recruited from purposefully selected government health offices within the region and invited to participate in key informant interviews. At each selected office, we invited one senior-level manager and one MNCH manager to participate in the study (except at PHCUs, where in the absence of MNCH managers, only senior-level managers participated). The interviews were semi-structured, allowing participants to respond in an uninhibited manner, while retaining a central focus on the topic of interest. In total, we conducted semi-structured interviews with 12 participants (1 female and 11 male) that held senior leadership, managerial or coordination positions at subnational levels of the health system in Ethiopia. These included directors, vice-directors, coordinators or MNCH focal points across regional (*n* = 2), zonal (n = 2), woreda (*n* = 5) and PHCU (*n* = 3) levels of administration. Interviews lasted 30–90 min and focused on 5 domains of questioning (Fig. 2).

The creation of interview guides was loosely informed by themes presented in two theoretical frameworks (an ecological framework of the social determinants of maternal and child health [30] and the framework for addressing equity through determinants of health [31]). The interview guide was pilot tested prior to data collection and revised for clarity and length. Investigation within the first domain (perceptions of relevant determinants of health) involved the use of a photo card showing a pregnant woman being carried on a traditional stretcher; participants were asked to comment on the acceptability and commonness of the scene, and underlying factors and conditions. To introduce the topic of health equity (domains 2–5), participants were read a description adapted from the World Health Organization (WHO) Commission on Social Determinants of Health [5]: “Health equity exists when everyone has a fair chance to achieve their full health potential. The opportunity to be healthy is available to everyone, regardless of their social, economic, demographic, or geographic characteristics.”

The interviews were conducted in November and December 2017 by one member of the research team, who had prior experience conducting semi-structured interviews, and doing research in the Ethiopian context. All interviews were conducted at a time and place that was convenient for the participant (typically the participant’s place of work). Participants were offered the option of doing the interview in English or in the local language of their choice with the assistance of an interpreter. Nine participants chose to do the interview in English, and three requested an interpreter for all or part of the interview. The interpreter, who has an ongoing relationship with the researchers, was briefed extensively about the study beforehand, and did verbatim, real-time translation [32]. All participants gave written informed consent to participate in the study and gave permission for their interview to be audio-recorded. The recordings were subsequently transcribed in written form. For interviews where an interpreter was present, the interpreter listened to the recording and reviewed the English transcript, making minor revisions where necessary.

Data were analysed through thematic analysis methods, using Atlas.ti software. Following multiple readings of the transcripts, a code guide was developed deductively based on the interview questions and expanded inductively to accommodate emergent concepts. Transcripts were coded, and cross-cutting themes were identified to illustrate understandings of health equity and perceived roles and responsibilities in addressing health inequities. Several researchers were involved in writing up the analysis. The findings of the study were discussed with experienced researchers working on related topics within the same zonal area, as well as national experts in the topic area. Researchers remained reflexive in identifying potential sources of bias and taking measures to limit them [33].

To ensure anonymity, the participants were assigned pseudonyms and are not identified by their job title or geographical location in the country; identifying details in participant quotes have been removed or altered.

## Results

### Understandings of health equity

Participants demonstrated detailed knowledge of government messaging about health equity, as several of them reiterated definitions or explanations of health equity that aligned closely with phrasing in Ethiopian Federal Ministry of Health documents; additionally, many participants linked the concepts of equity and quality, which are grouped together as one of three key features in the HSTP [7]. Participants all acknowledged that health equity was a concern within their respective jurisdictions. They provided multiple and diverse examples of health inequities that they had witnessed, which lend insight into how they understand health equity constructs.

### Health service delivery, quality and health sector adjacent factors

Participants expressed notions of ‘health’ that related to health service delivery, health service quality and health sector adjacent determinants. Most predominantly, participants provided examples of health inequity that pertained to health service delivery issues that fall within their scope of work as subnational health managers such as service accessibility, service use, funding/payment for health services, and the health workforce. Participants sometimes described health service delivery issues under the broad umbrella of “quality”, which they related to: the availability of materials, equipment and supplies; health worker training and professional conduct; health facility construction and cleanliness; and adherence to Ministry of Health standards and guidelines. Tedbabe^1^, for example, spoke about inequity in the availability of functional ambulances, which he explains, is linked to the ability to mobilize finances locally:

“We have a lot of budget approved from the government to put towards buying ambulances…. but it’s not enough to actually put ambulances in all woredas. Woredas are expected to have enough budget for the purchasing and also different maintenance issues and also for different operational costs associated with the ambulance.” -Tedbabe

Another participant noted that the supply of material resources at health centers differs based on the extent of support from non-governmental organizations (NGOs):

“Medical equipment, medicine, drugs: they impact on service quality. Some health centers are equipped by non-governmental organizations. The materials come from non-governmental organizations. The other health centers do not have the chance to get this material. So, there is a barrier. A difference in quality. In our [area], one health centre is [well supported] and three areas are also equipped very nicely because of the non-governmental support. The other health centers are [not because they are further away]. So, there is a difference.” -Tahir

In fewer instances, participants raised examples of ‘health’ that are adjacent to the health system, such as community mobilization and leadership for health, and as Ebise explains, susceptibility to disease:

“Because of different agricultural cultivation, there could be different scenarios. Like there are some areas that are highly cultivated and some areas that are not cultivated – there may be a difference. In those areas where there is a lack of agricultural cultivation, there may be malnutrition. And malnutrition may affect their [the local population] height.” -Ebise

### Geography and intersecting factors

Geographical dimensions featured prominently in how participants described the distribution of health; that is, health inequities were linked to having geographically dispersed populations with variable contexts and living conditions. While geography itself was considered important in characterizing health inequity, participants also explained how geography intersected with other factors. For instance, geographical remoteness combined with poor infrastructure (especially roads) was identified as a major barrier that affected transportation to health facilities, but also the ability of subnational health managers to provide training to health workers and perform supervisory activities. Mustafa, for instance, describes how the topography of an area is a determinant of health worker retention:

“[The catchment area] is very large, and the topography also makes it very hard to reach some areas… with this challenge, we are going to try to touch each kebele [community of 5000 people]. But there is one kebele that is very hard to reach: there is no road and as of now there is no HEW [Health Extension Worker].”^2^ -Mustafa

Health centers located in remote settings were also less likely to receive support from NGOs, while socio-cultural considerations, such as patriarchal gender norms, were sometimes expressed with a geographical dimension based on the presence or absence of them in certain areas:

“In most areas it exists that women are almost neglected and almost considered as a material. In some areas. They [women] are not decision makers, even about their own reproductive health issues. Even to get, for example – and this is a life or death issue – even to get skilled birth attendance they have to get their husbands to approve.” -Fikereye

An example of a distributional aspect of health inequity that did not have a geographical dimension identified social connectedness as a determinant of the quality of care received:

“Quality of care is not [equitable]. It may be varied from person to person…They [health workers] may give the one he knows very well good service. In other cases, it may be minimized.” -Tewdros

### Distinguishing between equity and equality

Participants described a range of perspectives surrounding the distinction between *equity* and *equality*. For instance, some participants expressed that addressing health inequities required looking at factors beyond *equal* distribution of health system resources. They problematized attempts to standardize aspects of the health system, explaining how standardization is not responsive to context-specific needs or circumstances from which inequities stem. Illustrating the distinction of potential coverage from actual coverage [34], Ayana described inequity as a “difference in service utilization among people while the services are equally available for all community members,” acknowledging that sometimes equality in health system delivery mechanisms does not reflect the varying realities between areas. Relatedly, he described why even an equal allocation of medications to all health centers is not enough to ensure their equitable distribution (which he attributes to inequalities in human resources):

“So, sometimes we received the drugs [back] from remote health centers. Initially we distributed the drugs for all health centers equally. But the community in the remote area may not get the services per their need, because the health professionals are not there to distribute those drugs.” -Ayana

In other cases, participants upheld that promoting health equity meant ensuring equality in service delivery irrespective of contextual factors that drive inequities. Najib, for instance, advocated for equality in the delivery of health services to all and the provision of MNCH services for free. For him, broader causes of health inequity (such as education level or place of residence) should not influence how services are provided:

“Since our aim is to give service to all mothers, we don’t ask them where they’re from. We are serving all mothers equally… And even if a mother, whether she came from a rural area or from the town, if she is educated or not educated, the service is free. And there is no basis for having any differences in how services are given. So, everything is equitable.” -Najib

### Advancing health equity

Building upon their expressed understandings of health equity, participants described how they approached advancing health equity. These approaches often were related to serving remote populations and strengthening operational aspects of the health system, but sometimes extended beyond this to address demand-side barriers to health service utilization (such as lack of knowledge or awareness), or broader determinants of health (such as gender inequities or lack of community leadership).

### Reaching underserved populations and low-performing areas

Foremost, all participants recognized that serving all people within their catchment area was an important aspect of their role as a subnational health manager. Many of these efforts centered on reaching geographically remote populations. For instance, Tahir described how, although health posts^3^ are designed to serve 5000 people, some health posts in remote areas could have catchment populations of up to 8000 people, spread over a vast area. To address this, Tahir explained how he, on his own initiative, used local budgets and fundraising to establish temporary health posts near remote villages; HEWs in these areas then worked out of multiple health posts (temporary and permanent). Tahir, whose efforts are not formally recognized by higher levels of the health system, commented, “By this method, we should get to equity.”

Tewdros explained how he promotes different approaches, depending on the setting and circumstances, to ensure that pregnant women arrive safely at health centers to deliver their babies. His recommendations are tailored based on the woman’s proximity to passable roads and ambulance accessibility.

“When labour happens they try to take her to the health centre. If there is any ambulance near to the household they may take her by using this. This is mainly if there is a road accessible. They take them to the road and they call the ambulance and the ambulance will give service to them… If there is no ambulance access, and if the road is not accessible, we recommend them to carry her using what we call a stretcher.” –Tewdros

### Strengthening health service delivery

Participants explained how, in many cases, their role in promoting health equity was to strengthen operational aspects of the health system such as maintaining an adequate and effective health workforce, ensuring availability of medications and services, and facilitating supportive supervision. Specific mechanisms to strengthen aspects of health service delivery mentioned by participants at the zonal level included: publicly recognizing both high- and low-performing areas; providing continuous training and encouragement to health workers; establishing transparent channels to receive and respond to public feedback; and hiring more health workers in remote areas where they are needed, and focusing on their retention.

Gali described how his team at the PHCU level lacked the resources to acquire transportation to perform supervisory visits to health posts in his catchment area. He explained how he pieced together resources (including contributions from his own salary) to address the issue:

“We have difficulty to go to the community because we have only one motorcycle. And that I see as a barrier… We do not have enough budget… We need transportation for [many reasons], and we need the money. We can make it work by [redirecting] a small amount of the overall budget or using our own salary to do this work. Because it has an impact on our work.” -Gali

According to participants, both positive public recognition and punitive measures were commonly used to promote improved health service delivery. While national Ministry of Health policies strongly discourage displays of disrespect or shaming towards patients (i.e. by health workers at the facility level) [7], subnational health offices routinely and openly engage in public shaming of under-performing areas. In detailing how his office encourages improvements of lower-level health offices, Fikereye explains:

“One thing, one initial thing is something that we do during the review meeting. We recognize some high performance [areas], and we prepare a sort of shame in low performing [areas]. This is one way.” -Fikereye

Likewise, another participant at the PHCU level described that health workers could sometimes be punished for not doing their job correctly or not following guidelines.

Participants frequently mentioned the importance of improving quality as a means to address health service inequities. They reported addressing quality issues through a variety of mechanisms such as making strategic budget allocations, increasing supportive supervision, and initiating explicit discussions about quality issues. Tariku explains how, in areas where the quality of antenatal care (ANC) is poor, he facilitates extra visits from health workers with the expectation of inciting quality improvements:

“Not all health facilities give equal ANC: one is very excellent and the other is very lazy… The community is going to the [better health facility]. Through time, we give supportive supervision to improve the [other] health facility. What do I mean? We assign a health worker to go to the low performing facility and support them…. They [the health workers performing supportive supervision] will go weekly.” -Tariku

Tahir, who works in a geographically large area, recognizes variable quality across communities in terms of health service delivery. He explains how connecting key stakeholders from the different communities encourages them to share their experiences, which in turn motivates them to advance service quality:

“The [catchment area] is huge… If all religious leaders and other community leaders came together to discuss this [health quality issue], they could learn about the experiences of the others… Why? [They know] how they teach the community, how they attract the community [to the facilities], how they engage the population to use the health services. Delivery is different from one [area] to the other. If they come together to discuss, one gets experience from the others. And this would improve the service quality.” –Tahir

### Addressing non-health determinants of health

Health equity was also addressed by participants through factors peripheral to the health system. While non-health factors were commonly considered in participants’ efforts to reach remote populations, strengthen health service delivery, and improve service quality (as detailed above), participants also described taking actions to directly address wealth- and gender-based inequalities that impacted health. Tedbabe, acknowledging urban-rural differences and wealth inequalities, spoke about the contribution of the health sector in providing extra support to rural areas and helping to address food security issues:

“We try to intervene in…rural areas to address differences based on wealth issues and so on. It’s another challenge that will not only be addressed from the health sector, but the overall goals of the country, really. …But in the health sector we have our programs and we are trying to give extra support to families that cannot afford to buy food and so on. We try to give priority for people who cannot afford these things. In certain areas they [food and health-related services] are provided for free.” -Tedbabe

For Fikereye, his role as a health manager included promoting female empowerment and encouraging greater participation of men in reproductive and maternal health issues. For instance, he encourages male partner involvement at medical appointments and community pregnancy forums.

“Now we, as government, are addressing [female] empowerment to make women equal to males… As part of the government we are getting the male partners to be involved in reproductive health issues and understand their partners and make decisions together… For example, when women come in to have HIV screening tests during pregnancy, we tell her to bring her husband for engagement in HIV partner tests. While the partner does the test we also discuss many issues about pregnancy… for example, [telling them] about things like her personal hygiene, and about basic care during pregnancy. Like maternal nutrition… At the community level there are different meetings, like the pregnancy forum hosted by midwives. Although the primary targets are pregnant women, if male engagement is needed we call for males and we make a discussion… we need the engagement of men to care for their wives and their partners.” -Fikereye

### Aligning with other sectors or groups

Participants described working with other government sectors, community groups, and NGOs to advance health equity. Work with community groups had a heavy focus on demand creation for health services, while NGO collaborations typically involved receiving education or training as well as material or equipment inputs. Participants also expressed having close ties with higher and lower levels of governance within the health sector.

#### Government sectors

Working with sectors like agriculture and education was raised by participants. While aligning with agriculture was a way to address hunger and malnutrition, the education sector was seen as important in promoting health literacy at the local scale, and ensuring high-quality training of health workers in adequate numbers. In some cases, these inter-sectoral collaborations were quite informal and ad hoc, such as sharing transportation to perform supervisory visits to remote areas; in other instances, these collaborations were institutionally formalized through programs focusing on, for example, nutrition or water, sanitation and hygiene.

One participant, Yared, provided details about how health and agriculture sectors work together, underscoring that when the population has poor agricultural outputs it strains the health system. He acknowledged that financial constraints limit the potential of these collaborations, as finances are needed to cover per diems and travel expenses.

#### Community groups

Another issue that surfaced in the interviews was how community groups were instrumental in efforts to promote health equity, and complementary to the work of the health sector. The Women Development Army (WDA), a cadre of community volunteers with a mandate to support the work of the HEWs at the local level, was mentioned frequently. Members of the WDA identify pregnant women to HEWs, who then follow up to provide ANC and to promote facility-based delivery. Najib describes how the WDA is very effective in parts of his catchment area – and less so in other parts: “If they work, really, they are very active. The problem is that normally they are not active and just staying home.”

For Mustafa, and several others, strengthening the WDA is a way to reach out to areas that do not presently have sufficient HEWs:

“The WDA is the closest to the community. We are strengthening their capacity as well as the creation of their awareness on health… this WDA training, which is to reduce maternal deaths, has reached around 84% of the WDA [in my catchment area] at this time.” -Mustafa

#### NGOs

Several participants described how contributions from NGOs were beneficial (especially in the health topics that were prioritized by the NGO) by helping to fill gaps in expertise, leadership, finances, and resources. Areas where NGOs worked benefited from an increased sense of commitment and community mobilization (e.g. through NGO-run programs that facilitate community mobilization). Several participants, however, discussed how the distribution of NGO contributions, while important, perpetuated geographical inequities. Tedbabe, who has interacted with NGOs in determining where they will work, described certain challenges that he encounters in trying to direct NGO engagement to remote areas:

“The challenge, for one, is the unfair distribution of the partners [NGOs]. It’s not fair that most of the partners are distributed by the towns and urban areas…usually they don’t want to go very far [outside of the cities]. We try to push them out into these areas actually – and they accept, but they don’t want to stay there. They come back. We are trying. Still, most of the NGOs and partners are in the central parts of the country, but we are managing to push them out a bit.” – Tedbabe

Tedbabe spoke about the challenge of aligning NGO priorities with the priorities of the Ethiopian health system, noting that NGOs may have interests and priorities that do not fit within the political agenda for health.

## Discussion

### Perceptions of health equity in Ethiopia

Ethiopia’s advancement on its global and national commitments to health equity relies on managers at subnational levels of the health system to realize improvements in equitable access to and delivery of quality health services. These improvements, in turn, rest partly on subnational health managers’ perspectives on health equity and how they can act to improve it. We are unaware of prior research probing this specific aspect of subnational health system responsibilities, hence undertook the study reported on in this article, as part of a larger implementation study [35–37].

Study participants easily recognized health inequities in their surroundings, which largely concerned issues embedded in the delivery of health services, and the geographical framing of health distribution. This finding is unsurprising, given that it reflects the administrative organization of the health system (hierarchically, across geographical divisions), and their mandate to increase health service coverage (see Table 1). Interestingly, geographical divisions were also the basis for comparing socio-cultural considerations such as gender norms. These findings add support to previous assertions that highlight the practical advantages of an area-based conceptualization of health inequity [38, 39], though as the authors of those studies caution, this can perpetuate ecological fallacies (that is, making undue assumptions about all individuals in an area based on population-level patterns).

The moral/ethical characterization of health distribution was conveyed through participants’ expressed unacceptability that the distribution of health disproportionally affected certain geographical areas, and their ongoing efforts to ameliorate this. Participants had a solid knowledge base about what was needed to improve the functioning of the health system in these areas, as stipulated in government standards and guidelines surrounding human resources for health, facilities and equipment, quality measures, etc. However, given the general lack of resources, the ability of subnational health managers to effect change was often limited, calling into question that the unequal health distribution was practically “unavoidable” – at least from the position of the subnational health managers. A previous study highlighting the differences across higher- and lower-performing PHCUs also noted resource and operational deficits in lower-performing facilities, which manifested in that study as: lack of data or mistrust of data quality; strained relationship between health facility staff, health workers and the community; and low contact and limited coordination with higher-level regulatory and financing bodies [40].

### Roles and responsibilities in tackling health inequities

While there is general agreement about the need for multipronged action to facilitate health equity gains through multiple entry points [5, 31], the role of the health system, and subnational health managers in particular, is less apparent. Baum (2007), like many others, advocates for top-down and bottom-up action on health equity (the “nutcracker effect”), calling for increased pressure from high-level policy makers and grassroots citizen groups, begging the question: what is the role for those in between?

In our study, subnational health managers expressed mixed views about their role in addressing causes of health inequities that extended beyond the health system. Some participants described taking initiative to address certain non-health factors, which often centered around transportation or access issues, though these tended to be more akin to ‘quick fixes’ than long-term solutions. Many of the mechanisms that subnational health managers used to advance health equity were applied inconsistently, relying on personal ingenuity and close familiarity with the populations and environments where they worked. Further, these mechanisms did not appear to be formally recognized or supported by the health sector in Ethiopia. Research in eastern Uganda reinforces the merits of encouraging subnational health managers to work creatively and flexibly to attain goals and collaborate with others [41], capacities that others suggest should be supported through institutional design and financing [13].

Shaming of under-performing subnational health offices emerged as part of a strategy intended to motivate performance improvements, as captured through select quantifiable outcome measures. The merit of stigmatization in public health has been questioned, as it places burden on those already in positions of social disadvantage [42]. Research in Ethiopia has explored the implications of shaming at the health facility level, finding that such approaches cause undue suffering for women, and may serve as a deterrent to service use [43]. In a similar way, we question how this approach might be non-facilitative at subnational levels of the health system. We highlight the possibility, for example, of obscuring the integrity of reporting practices, which are already known to be poorly coordinated and prone to quality concerns [44].

As part of their efforts to address health inequities, the subnational health managers in this study aligned their efforts with other sectors or groups in a variety of arrangements. These collaborations were valued (especially as a way to use resources more efficiently or to acquire new resources) though this sometimes introduced competing interests and unwanted complexities that led to exacerbated health inequities (notably, the role of NGOs in defining priority health topics, and geographical locales in which they were willing to provide resources). Indeed, coordination and collaboration with non-state actors is a recognized challenge across global health efforts [45], particularly since development partners may contribute substantially to health financing (in the case of Ethiopia, development partners contributed 15.30% of total health expenditures in 2015) [46]. Over the past two decades, the Ethiopian Federal Ministry of Health has benefited from strong leadership in rolling out certain donor coordination reforms [47]; however, our findings suggest a need to extend these efforts subnationally to ensure that NGO activities are better oriented to further health equity. Likewise, the scale-up of the WDA initiative should be informed by where gaps exist geographically, heeding the impact of the program design on the well-being of its participants [48].

### Decentralization in the health sector

Facing challenges when addressing health equity at the subnational governance level is not unique to Ethiopia. Other studies at the subnational level have previously found a general mismatch between policy responsibilities bestowed upon subnational governance actors and the financial resources provided to them for implementing an equitable policy agenda [49]. A similar criticism has long been raised in relation to the decentralization of health service more generally, which led to a rise in responsibility for lower level governance actors without matching (new) financial resources.

While decentralization was intended to improve operational efficiency while also contributing to more equitable health outcomes, in practice this has not been borne out. As one review study, which assessed decentralization across Latin American, African and Asian contexts, found: “the quality and equity of access have not improved with the decentralisation of health and education services; and equity and efficiency outcomes are closely related to the availability of financial resources and local government capacity” [50]. This mirrors some of our empirical findings which indicate that lack of fiscal capacity at the subnational governance level is a substantial impediment to achieving better health equity outcomes. At 5.98% of the government budget, health financing in Ethiopia remains, overall, below the Abuja target of 15% [46].

The challenges highlighted in our study speak to a larger issue, namely how to effectively coordinate governance actors and processes across multiple levels, in order to achieve better alignment between national policy priorities (such as Ethiopia’s HSTP), and subnational governance actions designed to achieve health equity. One particular concern in the context of Ethiopia’s resource scarcity is the accelerating capital flight (legal and illegal outflow of capital reaching over $US 1 billion annually) which undermines the ability of the central government to transfer resources to other government levels to achieve health equity-oriented policy objectives. This has led some to suggest that, in order to address health equity, more resources have to be mobilized domestically through progressive forms of taxation [51] (which, as of 2011, constituted 9.2% of GDP revenue in Ethiopia [46]). However, even if domestic resource mobilization could generate some of the much-needed, additional resources, acknowledging national level fiscal constraints to tackling health equity, others have explicitly called for international assistance for health as a way of strengthening equitable access to health service provision in the context of progressively implementing universal health coverage (UHC) [52, 53]. This could be achieved through a Global Fund to finance UHC which would transfer resources to national governments in low-income countries [54], which could then be used to allay the financial pressures experienced at the subnational level.

In this study, we explored how health equity is understood and pursued by subnational health managers across regional, zonal, woreda and PHCU levels in Ethiopia. We note that health system issues may resonate differently within each of these subnational levels, or in different regions across the country, as has been suggested in a previous comprehensive national assessment of all facilities that provide services for childbirth [55]. Our findings, while suggestive, should not be taken as generalizable across the whole of the Ethiopian health system.

### Implications and further research

Focusing on the three constructs of health equity – health, the distribution of health and the ethical/moral characterization of health distribution – allowed us to gain insight into how subnational health managers perceive and address health inequities. Subnational health managers are attuned to inequities, both within and adjacent to their work, and often rely on personal ingenuity to circumvent deficits in material, infrastructure, human and financial resources. By acknowledging, legitimizing, and supporting local solutions, the health sector can improve efficiencies, although localizing issues of distributional justice can lead to ignoring national or even global political and economic policies that exacerbate resource inequities far beyond the capabilities of local or subnational levels to mitigate [56]. Further explorations of such local solutions and means of facilitating knowledge sharing between subnational stakeholders within and between countries are warranted.

Geographical inequities in health are a prominent concern in Ethiopia and indeed, a major ongoing challenge for the health sector in Ethiopia lies in extending high quality, essential health services to rural and remote populations [7, 36]. However, the meaning of “health equity” is likely to evolve over time as its three constructs noted above shift to reflect changing contexts and priorities. Pragmatic approaches have been developed to help governments navigate the practical integration of health equity considerations into national health policies and/or policy-making processes, using participatory approaches to ensure that diverse stakeholder perspectives are captured [57, 58]. Interestingly, our findings suggest subnational health managers to be very familiar with government messaging around health equity, and therefore policy documents and strategies are promising opportunities to harmonize understandings and establish norms surrounding the advancement of health equity. But, given that one of the major limitations to improving health equity at the subnational governance level is resource endowment, an important consideration going forward will be to ensure that expectations about health equity oriented policy actions at the subnational level are matched with appropriate financing, so as to avoid the decentralization of responsibilities without matching resources.

## Footnotes

1 All participants were assigned pseudonyms to protect their anonymity.

2 Health Extension Workers (HEWs) are locally-recruited, female community health workers that are paid to deliver disease prevention and health promotion activities at the kebele (village) level, predominantly through community mobilization and health education.

3 Health posts are the most decentralized level of health facility, designed to serve kebeles of 5000 people and be staffed by at least two HEWs. Strategies to account for kebeles that have more (or less) than 5000 people are not uniform across the country [59].
